# Genome Sequence of *Escherichia coli* E28, a Multidrug-Resistant Strain Isolated from a Chicken Carcass, and Its Spontaneously Inducible Prophage

**DOI:** 10.1128/genomeA.00348-17

**Published:** 2017-06-01

**Authors:** Imke Schmidt, Thomas Riedel, Isabel Schober, Boyke Bunk, Cathrin Spröer, Anna Bierbrodt, Hansjörg Lehnherr, Johannes Wittmann

**Affiliations:** a Leibniz Institute DSMZ-German Collection of Microorganisms and Cell Cultures, Braunschweig, Germany; b FINK TEC GmbH, Hamm, Germany; c PTC Phage Technology Center GmbH, Bönen, Germany

## Abstract

In this study, we sequenced the complete genome of the multidrug-resistant *Escherichia coli* strain E28, which was used as an indicator strain for phage therapy *in vivo*. We used a combination of single-molecule real-time and Illumina sequencing technology to reveal the presence of a spontaneously inducible prophage.

## GENOME ANNOUNCEMENT

Antibiotic resistance in *Escherichia coli* is of particular concern, as it is one of the most common Gram-negative pathogens in humans and animals. As *E. coli* can be transmitted via contaminated food, it is of special interest to establish alternatives to antibiotic treatment to prevent the spread of resistances (1).

The strain E28 was isolated from a chicken carcass in Germany in 2012 and preexamined with *E. coli* PanType (Alere Technologies GmbH) and the phenotypic assay Gen III MicroPlate (Biolog), whereby it was possible to identify the virulence markers *astA* (heat-stable enterotoxin 1 gene), *prfB* (P-related fimbriae regulatory gene), *ireA* (iron-regulated gene), *hemL* (glutamate 1-semialdehyde aminotransferase gene), and the resistance to potassium tellurite and kanamycin potassium tellurite and kanamycin. It was defined as an extended-spectrum β-lactamase-producing strain (*bla*_CTX-M-9_) and characterized as serotype H34.

In this study, we determined *de novo* the complete genome sequence of *E. coli* strain E28 (=DSM 103246) using a combination of single-molecule real-time (SMRT) and Illumina sequencing technologies. The strain was cultivated aerobically in tryptone soya broth (Thermo Scientific, Oxoid) at 37°C. For extraction of genomic DNA, the genomic-tip 100/G kit (Qiagen) was used according to the manufacturer’s instructions. P6 chemistry was used to perform genome sequencing on the PacBio RSII platform (Pacific Biosciences). The genome was assembled with the “RS_HGAP_Assembly.3” protocol included in SMRT Portal version 2.3.0, utilizing 138,634 postfiltered reads with an average read length of 12,044 bp. The assembly yielded one complete chromosomal contig, which was circularized and adjusted to *dnaA* (Eco28_00001) as the first gene. Complementary genome sequencing was carried out on a MiSeq (Illumina) in a 75-bp paired-end double-indexed run, resulting in 6.73 million paired-end reads. Library preparation was conducted as previously described (2). The Burrows–Wheeler aligner (3) was used for quality improvement of the final consensus sequence by mapping the Illumina reads onto the obtained contig. A final quality of QV60 was confirmed. Prokka was used for automated genome annotation (4).

Genome sequencing of *E. coli* strain E28 resulted in a complete genome of 5,445,834 bp with a G+C content of 50.51%, which contains 5,058 predicted coding sequences, 22 rRNAs, and 86 tRNAs. Analysis with ResFinder (5) revealed several genes that putatively mediate resistance against antibiotics: *aph(3′')-Ib* (Eco28_04784), *aph(six)-Id* (Eco28_04785), *aph*_*(3′)*_*-Ic* (Eco28_04786), *aadA2* (Eco28_05120), *blaCTX-M-14b* (Eco28_05116), *sul2* (Eco28_04812), *sul1* (Eco28_05118), *tet*_*(B)*_ (Eco28_04800), and *dfrA16* (Eco28_05121). Using PHASTER (6), we identified several putatively intact prophage regions (Pro1: Eco28_01223-Eco28_01239, Pro2: Eco28_01264-Eco28_01314, Pro3: Eco28_01418-Eco28_01475, Pro4: Eco28_02472-Eco28_02548, Pro5: Eco28_02723-Eco28_02788, Pro6: Eco28_03037-Eco28_03084, Pro7: Eco28_04580-Eco28_04628, and Pro 8: Eco28_05122-Eco28_05161) with sequence similarities to, for example, phage P4 (7) or P88 (GenBank accession no. KP063541) in the genome. Interestingly, at least one of these prophages was shown to be spontaneously inducible since genome sequencing revealed a second contig after sequence assembly that matched exactly to bases 1419048 to 1460607 of E28, representing prophage region P3.

### Accession number(s).

The complete genome has been deposited at GenBank under the accession number CP019944. The version described in this paper is the first version, CP019944.1.

## References

[1] European Centre for Disease Prevention and Control 2017 Antimicrobial resistance surveillance in Europe 2015. *In* Annual Report of the European Antimicrobial Resistance Surveillance Network (EARS-Net). European Centre for Disease Prevention and Control, Stockholm, Sweden.

[2] Baym M, Kryazhimskiy S, Lieberman TD, Chung H, Desai MM, Kishony R 2015 Inexpensive multiplexed library preparation for megabase-sized genomes. PLoS One 10:e0128036. doi:10.1371/journal.pone.0128036. 26000737 PMC4441430

[3] Li H, Durbin R 2010 Fast and accurate long-read alignment with Burrows-Wheeler transform. Bioinformatics 26:589–595. doi:10.1093/bioinformatics/btp698. 20080505 PMC2828108

[4] Seemann T 2014 Prokka: rapid prokaryotic genome annotation. Bioinformatics 30:2068–2069. doi:10.1093/bioinformatics/btu153. 24642063

[5] Zankari E, Hasman H, Cosentino S, Vestergaard M, Rasmussen S, Lund O, Aarestrup FM, Larsen MV 2012 Identification of acquired antimicrobial resistance genes. J Antimicrob Chemother 67:2640–2644. doi:10.1093/jac/dks261. 22782487 PMC3468078

[6] Arndt D, Grant JR, Marcu A, Sajed T, Pon A, Liang Y, Wishart DS 2016 PHASTER: a better, faster version of the PHAST phage search tool. Nucleic Acids Res 44:W16–W21. doi:10.1093/nar/gkw387. 27141966 PMC4987931

[7] Halling C, Calendar R, Christie GE, Dale EC, Dehò G, Finkel S, Flensburg J, Ghisotti D, Kahn ML, Lane KB 1990 DNA sequence of satellite bacteriophage P4. Nucleic Acids Res 18:1649. doi:10.1093/nar/18.6.1649. 2183201 PMC330555

